# The alternative lengthening of telomeres mechanism jeopardizes telomere integrity if not properly restricted

**DOI:** 10.1073/pnas.2208669119

**Published:** 2022-09-19

**Authors:** Bruno Silva, Rajika Arora, Claus M. Azzalin

**Affiliations:** ^a^Instituto de Medicina Molecular João Lobo Antunes, Faculdade de Medicina, Universidade de Lisboa, Lisboa 1649-028, Portugal

**Keywords:** alternative lengthening of telomeres, telomere transcription, TERRA, Mus81

## Abstract

A substantial number of human cancers are telomerase-negative and elongate physiologically damaged telomeres through a break-induced replication (BIR)-based mechanism known as alternative lengthening of telomeres (ALT). We recently demonstrated that inhibiting the transcription of the telomeric long noncoding RNA TERRA suppresses telomere damage and ALT features, indicating that telomere transcription is a main trigger of ALT activity. Here we show that experimentally increased TERRA transcription not only increases ALT features, as expected, but also causes rapid loss of telomeric DNA through a pathway that requires the endonuclease Mus81. Our data indicate that the ALT mechanism can endanger telomere integrity if not properly controlled and point to TERRA transcription as a uniquely versatile target for therapy.

Alternative lengthening of telomeres (ALT) cancers achieve immortality by reelongating their telomeres in the G2 and M phases of the cell cycle through a specialized break-induced replication (BIR) pathway (1, 2). The toxic nature of the ALT mechanism was previously proposed based on correlative evidence. We and others showed that ALT telomeres are highly transcribed and enriched in RNA:DNA hybrids (telR loops) (3–5). Depletion of the endoribonuclease RNAseH1 or the translocase FANCM in ALT cells increases telR loops, ALT activity, and telomere instability. We thus suggested that ALT might compromise telomere integrity if not properly restricted (3, 4). However, this proposal has remained speculative because the telomeric defects observed in RNaseH1- and FANCM-depleted cells may derive from indirect effects associated with depleting proteins that do not exclusively act at telomeres.

By employing transcription activator-like effectors targeting telomeric repeat-containing RNA (TERRA) promoters comprising specific CpG-rich, 29-bp repeats (T-TALEs), and fused to a transcription suppression domain, we demonstrated that inhibiting TERRA transcription alleviates ALT activity (6). This discovery, by establishing that TERRA transcription is a major trigger of ALT, prompted us to assess the consequences of triggering excessive ALT.

## Results and Discussion

**Fig. 1.**
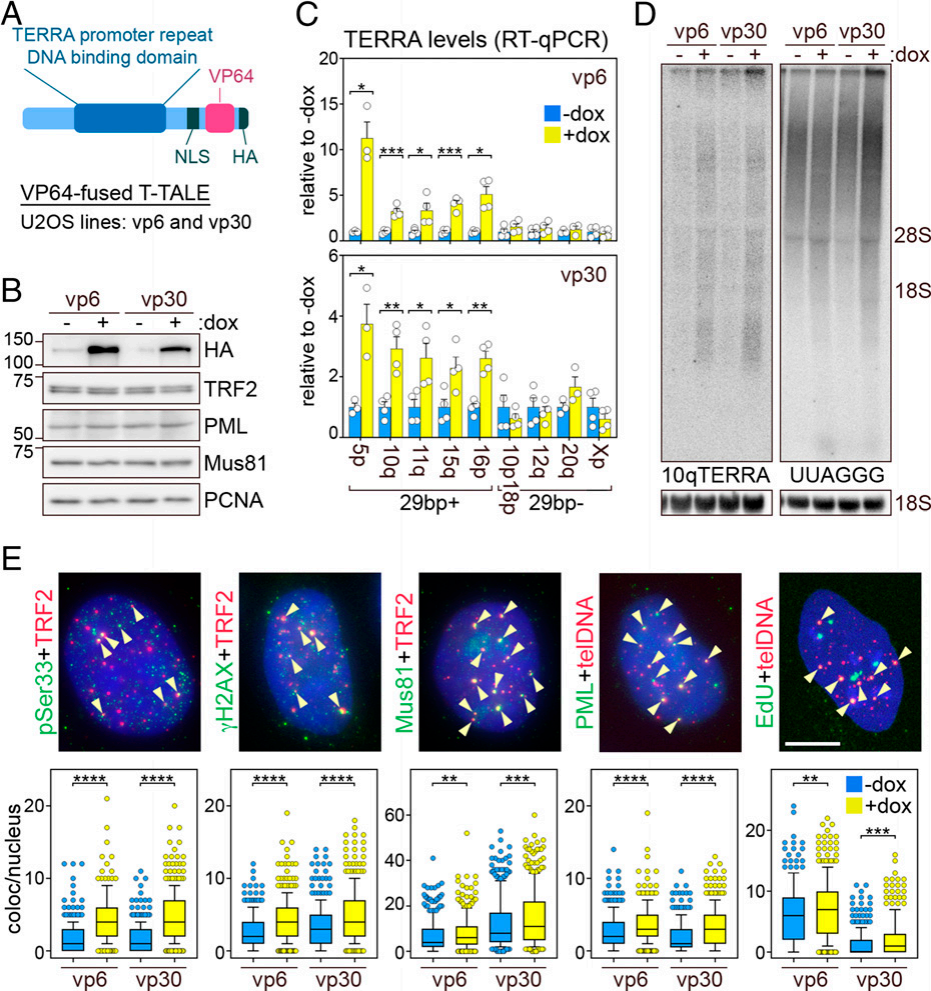
(*A*) Schematic representation of T-TALEs. (*B*) Western blot of T-TALEs (anti-HA antibody) and proteins analyzed throughout the study. Cells were treated with dox for 24 h. PCNA is the loading control. (*C*) The qRT-PCR quantifications of TERRA transcripts from 29-bp-containing (+) and 29-bp-devoid (−) subtelomeres in cells as in *B*. Values are normalized to −dox. Bars and error bars are means and SEMs from three or four independent experiments. Circles are single data points. Asterisks are *P* values (two-tailed Student’s *t* test). (*D*) Northern blot of 10qTERRA or total UUAGGG repeats in cells as in *B*. The positions of 28S and 18S ribosomal RNAs (rRNAs) are on the right. The 18S rRNA hybridization is the loading control. (*E*) Examples of pSer33, γH2AX, Mus81, and PML indirect immunofluorescence (IF) or EdU click-it staining (green) combined with TRF2 IF or telomeric DNA FISH (red) in cells as in *B*. Nuclear DNA is in blue. Arrowheads point to colocalization events. (Scale bar, 10 μm.) Box plots represent the 10th to 90th percentile of colocalization events per nucleus. Central lines are medians. At least 306 nuclei from three independent experiments were analyzed for each sample. Asterisks are *P* values (Mann–Whitney *U* test). *P < 0.05, **P < 0.005, ***P < 0.001, ****P < 0.0001.

We generated a T-TALE C-terminally fused to a nuclear localization signal (NLS), the RNA polymerase II transcriptional activator VP64, and a human influenza hemagglutinin (HA) epitope (Fig. 1*A*). The transgene was cloned downstream of a doxycycline (dox) inducible promoter and used to generate two independent ALT, U2OS-derived clonal cell lines dubbed vp6 and vp30 (Fig. 1*A*). Inducible transgene expression was validated by Western blotting using anti-HA antibodies (Fig. 1*B*).

We treated cells with dox for 24 h and performed TERRA qRT-PCR. TERRA transcripts from 29-bp-containing subtelomeres were 3- to 10-fold higher than in untreated cells, while TERRA from 29-bp-devoid subtelomeres remained unchanged (Fig. 1*C*). Northern blot hybridization with probes detecting TERRA from the 10q subtelomere or (UUAGGG)n sequences comprised in all TERRA molecules showed an increase in TERRA species upon dox treatment (Fig. 1*D*). Hence, our system can efficiently enhance TERRA transcription in U2OS cells.

We then monitored the accumulation of the replication stress marker RPA32 phosphorylated at serine 33 (pSer33) and of the general DNA damage marker histone H2AX phosphorylated at serine 139 (γH2AX) at telomeres. We also monitored ALT by quantifying ALT-associated PML bodies and events of de novo synthesis of telomeric DNA in G2 phase by EdU incorporation. In agreement with TERRA transcription promoting telomere instability and ALT, the incidence of all features increased in cells treated with dox (Fig. 1*E*). The observed effects truly derive from increased telomere transcription, as we previously showed that dox treatments did not affect TERRA levels, telomere stability, and ALT in control cells (nls1) that express T-TALEs not fused to transcription regulatory domains (6).

**Fig. 2.**
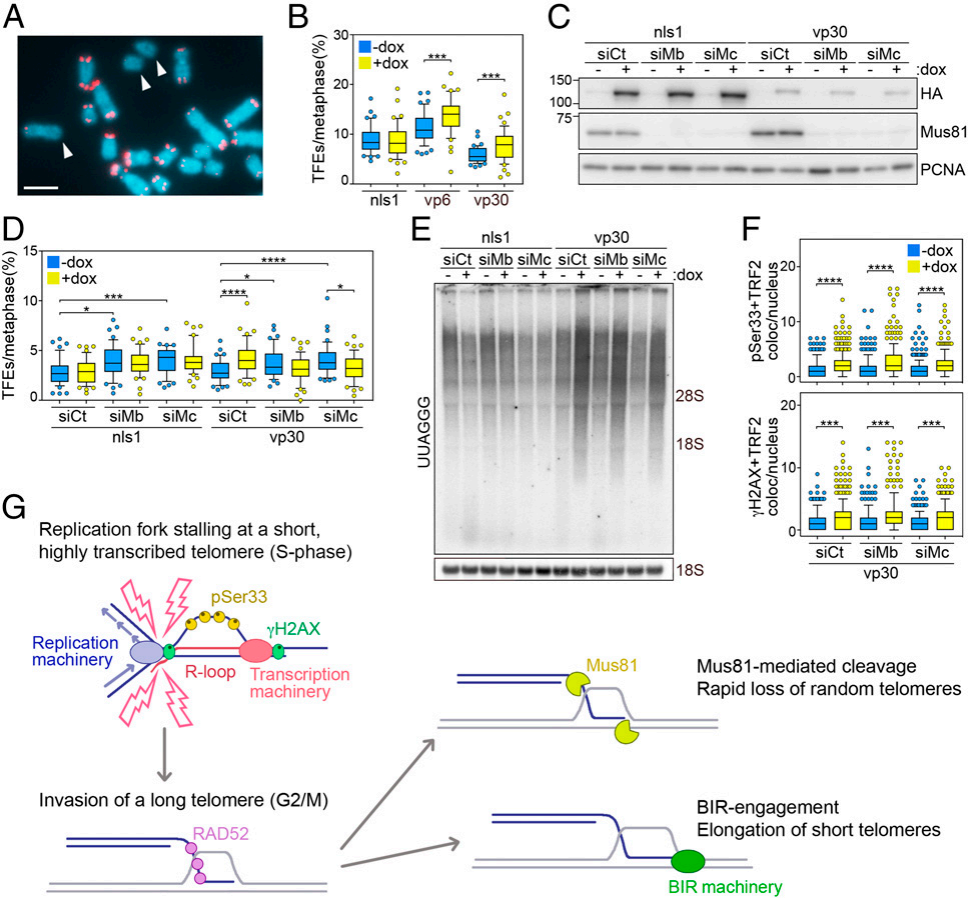
(*A*) Example of telomeric DNA FISH utilized to score TFEs (arrowheads). Chromosomes are from dox-treated vp30 cells. Telomeric DNA is in red; chromosomal DNA is in blue. (Scale bar, 5 μm.) (*B*) Box plots of the 10th to 90th percentile of TFEs per metaphase in cells treated with dox for 9 d. (*C*) Western blot of T-TALE (HA) and Mus81 levels in cells transfected with Mus81 (siMb and siMc) or control siRNAs (siCt). Cells were harvested 78 h after transfection. Where indicated, dox was included in the medium 6 h after transfection and remained until harvesting. PCNA is the loading control. (*D*) Box plots of the 10th to 90th percentile of TFEs per metaphase in cells as in *C*. For graphs in *B* and *D*, at least 2,596 chromosomes from three independent experiments were analyzed for each condition. (*E*) Northern blot of total UUAGGG repeats in cells as in *C*. The positions of 28S and 18S rRNAs are on the right. The 18S rRNA hybridization is the loading control. (*F*) Box plots of the 10th to 90th percentile of events of colocalization of pSer33 or γH2AX with TRF2 per nucleus in cells as in *C*. At least 303 nuclei from three independent experiments were analyzed for each sample. For all box plots, central lines are medians, and *P* values (Mann–Whitney *U* test). *P < 0.05, ***P < 0.001, ****P < 0.0001. (*G*) Model for the ALT mechanism (see text for details). Pink lightning denotes replication stress.

TERRA transcription inhibition increases the frequencies of chromosome ends devoid of detectable telomeric DNA signals (telomere free ends [TFEs]) in metaphase DNA fluorescence in situ hybridization (FISH) experiments (6). This is consistent with TERRA transcription inhibition alleviating ALT and causing progressive telomere shortening. Surprisingly, TFEs accumulated also in vp6 and vp30 cells treated with dox for 9 d, while no effect was observed in nls1 cells (Fig. 2 *A* and *B*). This suggests that a telomere loss mechanism, different from replicative shortening, is activated upon increased telomere transcription.

The structure-specific endonuclease Mus81 associates with ALT telomeres (7), and dox treatments increased the number of telomeric Mus81 foci in vp6 and vp30 cells (Fig. 1*E*). We depleted Mus81 using two independent small interfering RNAs (siRNAs) in nls1 and vp30 cells (Fig. 2*C*) and analyzed TFEs again. Consistent with previous work (7), Mus81 depletion increased TFEs in nls1 and vp30 cells not treated with dox (Fig. 2*D*). Importantly, Mus81 depletion prevented TFE accumulation when vp30 cells were treated with dox (Fig. 2*D*), without averting TERRA transcription induction or accumulation of pSer33 and γH2AX at telomeres (Fig. 2 *E* and *F*). Hence, Mus81 is largely responsible for the telomere loss events associated with increased TERRA transcription. The fact that depleting Mus81 in dox-treated vp30 cells suppressed TFEs but not telomeric γH2AX foci suggests that several TFEs are not yet deprotected and that, in our assays, γH2AX is prevalently marking arrested telomeric replication forks rather than double-stranded breaks (8).

Based on our work and data from other laboratories, we propose a model for ALT initiating with a highly transcribed, shortened telomere (9, 10) experiencing replication stress deriving from conflicts between the transcription and replication machineries (Fig. 2*G*). Ensuing telR loops might contribute to the amplification of the stress, possibly by slowing down the transcription machinery and/or directly hindering replication (11). Conflicts likely occur in S phase, when telomeres are replicated (12) and the ATR kinase generates pSer33 and γH2AX at stalled replication forks (8, 13). In the following G2/M phase, the G overhang of the damaged telomere invades a donor telomere in a reaction requiring RAD52 and involving the formation of a displacement-loop intermediate (14), which can engage in one of two alternative and competing reactions: On the one side, the assembly of an ALT-specific replisome allows BIR initiation and elongation of the shortened telomere (2); on the other side, Mus81 cleaves the displacement loop (15), thereby resolving the recombination intermediate and, eventually, shortening the donor telomere (Fig. 2*G*).

Our work establishes, in a direct manner, that the ALT mechanism involves molecular events with the potential to endanger telomere stability. Therefore, ALT has to be kept under control to allow telomere elongation and indefinite cell proliferation without excessive telomere loss. We also propose that TERRA transcription represents an exceptionally versatile target for ALT cancer therapy; indeed, both increasing and decreasing TERRA transcription should hinder cancer cell proliferation by compromising telomere maintenance.

## Materials and Methods

T-TALE-expressing U2OS clones were generated as previously described (6) and maintained in high-glucose Dulbecco’s modified Eagle’s medium GlutaMAX supplemented with 10% fetal bovine serum. When indicated, 50 ng/mL dox was added to the culture medium. All experimental procedures were previously described (6). Materials and protocols are detailed in *SI Appendix*.

## Supplementary Material

Supplementary File

## Data Availability

All study data are included in the article and/or *SI Appendix*. Materials will be made available upon resonable request.
