# Supplementary material for: The alternative lengthening of telomeres mechanism jeopardizes telomere integrity if not properly restricted
Source: Proc Natl Acad Sci U S A. 2022 Sep 19;119(39):e2208669119. doi: 10.1073/pnas.2208669119 (PMC9522348; doi:10.1073/pnas.2208669119)
Supplement: Supplementary File [file pnas.2208669119.sapp.pdf]

The Alternative Lengthening of Telomeres mechanism jeopardizes telomere integrity if not properly restricted

Supporting Appendix SI

Extended Materials and Methods

Plasmid construction

T-TALE cDNA was generated as previously described (1) and cloned into a KpnI/ApaI digested pcDNA5-FRT-TO plasmid (ThermoFisher Scientific) downstream of a doxycycline-inducible CMV promoter (unfused T-TALEs used for control experiments). The obtained plasmid was digested with ClaI and EcoRV and ligated to a fragment comprising a transcriptional activator domain composed of four copies of the Herpes Simplex Viral protein 16 and synthesized at GenScript (VP64 T-TALE). Plasmid sequences are available upon request.

Cell culture procedures

T-TALE expressing cells were generated by FRT-mediated integration of unfused T-TALE and VP64 T-TALE plasmids into T-REx™-U2OS cells expressing the TetR protein (ThermoFisher Scientific). Clonal selection was performed by plating cells at low dilution in high glucose DMEM, GlutaMAX (Thermo Fisher Scientific) supplemented with 10% tetracycline-free fetal bovine serum (Pan BioTech), 100 U/ml penicillin-streptomycin (Thermo Fisher Scientific) and 200 μg/ml hygromycin B (VWR). Individual clones were manually picked and expanded in the same medium. For T-TALE induction, 50 ng/ml doxycycline (dox; Sigma-Aldrich) was added to the culture medium devoid of hygromycin B for 24-72 h; for longer induction times, dox was refreshed every 72 h. Mycoplasma contaminations were tested using the LookOut Mycoplasma PCR Detection Kit (Sigma-Aldrich) according to the manufacturer's instructions. For Mus81 depletion, siRNAs were purchased from Integrated DNA Technologies and transfected at 30 nM concentration using the Lipofectamine RNAiMAX reagent (Invitrogen). Target sequences were: siCt (control): 5'-AUACGCGUAUUAUACGCGAUUAAC-3'; siMb: 5'-GAAGAGCAUGGUUCCGUCCAC-3'; siMc: 5'-UGUCACCAACACUCAGGUCAU-3'.

Western blotting

Cells were trypsinized and pelleted by centrifugation at 500 *g* at 4°C for 5 min. Pellets were resuspended in 2x lysis buffer (4% SDS, 20% Glycerol, 120 mM Tris-HCl pH 6.8), boiled at 95°C for 5 min and centrifuged at 1600 *g* at 4°C for 10 min. Supernatants were recovered and protein concentrations determined using a NanoDrop 2000 (ThermoFisher Scientific™). 30 μgs of proteins were mixed with 0.004% Bromophenol blue and 1% β-Mercaptoethanol (Sigma-Aldrich), incubated at 95°C for 5 min, separated in polyacrylamide gels, and transferred to nitrocellulose membranes (Maine Manufacturing, LLC) using a Trans-Blot SD Semi-Dry Transfer Cell apparatus (Bio-Rad). The following primary antibodies were used: a rabbit monoclonal anti-HA (Cell Signaling, 3724; 1:1000 dilution), a mouse monoclonal anti-PCNA (Santa Cruz Biotechnology, sc-56; 1:10000), a rabbit polyclonal anti-TRF2 (Novus Biologicals, NB110-57130; 1:2000), a mouse monoclonal anti-PML (Santa Cruz Biotechnology, sc-966; 1:500), a mouse monoclonal anti-Mus81 (Santa Cruz Biotechnology, sc-47692; 1:500). Secondary antibodies were HRP-conjugated goat anti-mouse (Bethyl Laboratories, A90-116P; 1:3000) and anti-rabbit (Bethyl Laboratories, A120-101P; 1:3000) IgGs. Chemiluminescence signals were acquired using an Amersham 680 blot and gel Imager.

RNA preparation and analysis

Total RNA was isolated using the TRIzol reagent (Thermo Fisher Scientific) followed by chloroform extraction and treated three times with 3.5 U of DNaseI (Qiagen) for 45 minutes at room temperature. For RT-qPCR, 5 μg of RNA were reverse transcribed with 0.5 μM TeloR (5'-CCCTAACCCTAACCCTAACCCTAACCCTAA-3') and 0.05 μM ActinR oligonucleotides (5'-AGCACTGTGTTGGCGTACAG-3') and Superscript III (Thermo Fisher Scientific) according to the manufacturer's instructions. Quantitative PCRs were performed using the iTaq Universal SYBR Green Supermix (Bio-Rad) on a Rotor-Gene Q (Qiagen) instrument with a 2-step program (45 cycles of denaturation at 95°C for 15 sec, annealing and extension at 60°C for 30 sec). Oligonucleotide sequences were: 5'-GAGTGCATTAGCATACAGGTG-3' and 5'-TCCTAATGCACACGTAACAC-3' for 5pTERRA, 5'-GAATCCTGCGCACCGAGAT-3' and 5'-CTGCACTTGAACCCTGCAATAC-3' for 10qTERRA, 5'-CTGATTATTCAGGGCTGCAAA-3' and 5'-GCCGCATCGACGGTGAATAA-3' for 11qTERRA, 5'-CAGCGAGATTCTCCCAAGCTAAG-3' and 5'-AACCCTAACCACATGAGCAACG-3' for 15qTERRA, 5'-TGTGTTTCAACGCTGCAACTG-3' and 5'-AGTTAGAACGGTTCAGTGTG-3' for 16pTERRA, 5'-CCTTCTAACTGGACTCTGAC-3' and 5'-

GCCACAGCGACGGTAAATAA-3’ for 10p18pTERRA, 5’-ATTTCCCGTTTTCCACACTGA-3’ and 5’-CTGTTTGCAGCGCTGAATATTC-3’ for 12qTERRA, 5’-GCAGCTTTCTCAGCACAC-3’ and 5’-TTTGTTCACTGTCGATGCG-3’ for 20qTERRA, 5’-GCAAAGAGTGAAAGAACGAAGCTT-3’ and 5’-CCCTCTGAAAGTGGACCAATCA-3’ for XpTERRA, 5’-TCCCTGGAGAAGAGCTACGA-3’ and 5’-AGCACTGTGTTGGCGTACAG-3’ for beta Actin. Data analysis was performed using the Rotor-Gene 6000 Series Software 1.7. Actin values were used as normalizers. For northern blotting, 10 μgs of RNA were separated in 1.2% agarose gels containing 0.7% formaldehyde and transferred onto nylon membranes. Gels were incubated with 50mM NaOH, 1.5M NaCl for 10 min at room temperature prior to transfer. To detect total TERRA (UUAGGG pool), RNA was hybridized at 55°C overnight with a double-stranded telomeric probe (Telo2 probe (1)) radioactively labeled using Klenow fragment (New England Biolabs) and [α-^{32}P]dCTP. Post-hybridization washes were twice in 2x SSC, 0.2% SDS for 20 min and once in 0.1x SSC, 0.2% SDS for 30 min at 55°C. To detect 10qTERRA, RNA was hybridized at 65°C overnight with a double-stranded 10q subtelomeric probe radioactively labeled using Klenow fragment (New England Biolabs) and [α-^{32}P]dCTP and [α-^{32}P]dGTP. Post-hybridization washes were twice in 2x SSC, 0.2% SDS for 20 min and once in 0.2x SSC, 0.2% SDS for 30 min at 60°C. After radioactive signal acquisition, membranes were stripped and re-hybridized at 55°C overnight with a 18S rRNA oligonucleotide (5’-CCATCCAATCGGTAGTAGCG-3’) radioactively labeled using T4 Polynucleotide Kinase (New England Biolabs) and [γ-^{32}P]ATP. Post-hybridization washes were twice in 2x SSC, 0.2% SDS for 20 min and once in 1x SSC, 0.2% SDS for 30 min at 50°C. Radioactive signals were detected using a Typhoon FLA 9000 imager (GE Healthcare) and quantified using ImageJ software.

DNA fluorescence *in situ* hybridization (FISH)

Cells were incubated with 200 ng/ml Colchicine (Sigma-Aldrich) for 5 h and mitotic cells were harvested by shake-off and incubated in 0.075 M KCl at 37 °C for 10 min. Chromosomes were fixed in ice-cold methanol/acetic acid (3:1) and spread on glass slides. Slides were then treated with 20 μg/ml RNase A (Sigma-Aldrich), in 1x PBS at 37 °C for 1 h, fixed in 4% formaldehyde (Sigma-Aldrich) in 1x PBS for 2 min, and treated with 70 μg/ml pepsin (Sigma-Aldrich) in 2 mM glycine, pH 2 (Sigma-Aldrich) at 37°C for 5 min. Slides were fixed again with 4% formaldehyde in 1x PBS for 2 min, incubated subsequently in 70%, 90% and 100% ethanol for 5 min each, and air-dried. A C-rich telomeric PNA probe (5’-AF568-OO-

CCCTAACCCTAACCCTAA-3'; Panagene) diluted in hybridization solution (10 mM Tris-HCl pH 7.2, 70% formamide, 0.5% blocking solution (Roche)) was applied onto the slides followed by incubation at 80°C for 5 min and at room temperature for 2 h. Slides were washed twice in 10 mM Tris-HCl pH 7.2, 70% formamide, 0.1% BSA and three times in 0.1 M Tris-HCl pH 7.2, 0.15 M NaCl, 0.08% Tween-20 at room temperature for 10 min each. DNA was counterstained with 100 ng/ml DAPI (Sigma-Aldrich) in 1x PBS and slides were mounted in Vectashield (Vectorlabs). Images were acquired with a Zeiss Cell Observer equipped with a cooled Axiocam 506 m camera and a 63X/1.4NA oil DIC M27 PlanApo N objective. Image analysis was performed using ImageJ and Photoshop software. TFEs were identified as chromatid ends lacking a detectable telomeric signal and were scored as: (TFE number/chromatid end number) x 100.

EdU incorporation and detection at telomeres

Cells grown on coverslips were incubated in medium containing 2 mM Thymidine (Sigma-Aldrich) for 21 h before replacement with fresh dox-containing medium. After 4 h, 10 μM RO-3306 (Selleckchem) was added and 18 h later 10 μM EdU (Thermo Fisher Scientific) was added to the culture medium, followed by a 2.5 h incubation. Cells were hybridized as for DNA FISH, washed twice with 1x PBS and EdU was detected using the Click-iT EdU Alexa Fluor 488 Imaging Kit (Thermo Fisher Scientific) according to the manufacturer's instructions. DNA was counterstained with 100 ng/ml DAPI in 1x PBS and coverslips were mounted on slides in Vectashield. Image acquisition and analysis were as for DNA FISH. Synchronization in G2 was controlled by propidium iodide staining followed Flow cytometry analysis performed on a BD Accuri C6 (BD Biosciences).

Indirect immunofluorescence (IF)

Cells grown on coverslips were washed twice in ice-cold 1x PBS and incubated in CSK buffer (100 mM NaCl, 300 mM sucrose, 3 mM $MgCl_2$, 0.5% Triton X-100, 10 mM PIPES pH 7) for 7 min on ice, fixed with 4% formaldehyde (Sigma-Aldrich) in 1x PBS for 10 min and permeabilized again with CSK buffer for 5 min. Fixed cells were incubated in blocking solution (0.5% BSA, 0.1% Tween-20 in 1x PBS) for 1 h followed by incubation in blocking solution containing primary antibodies for 1 h, three washes with 0.1% Tween-20 in 1x PBS for 10 min each, and incubation with secondary antibodies diluted in blocking solution for 50 min. For combined IF and DNA FISH, cells were again fixed with 4% formaldehyde in 1x PBS for 10 min,

washed three times with 1x PBS, incubated in 10 mM Tris-HCl pH 7.2 for 5 min and then denatured and hybridized with a PNA probe as for DNA FISH. DNA was counterstained with 100 ng/ml DAPI in 1x PBS or in 0.1 M Tris-HCl pH 7.2, 0.15 M NaCl, 0.08% Tween-20. Coverslips were mounted on slides in Vectashield. The following primary antibodies were used: a mouse monoclonal anti-TRF2 (Millipore, 05-521; 1:2000), a mouse monoclonal anti-PML (Santa Cruz Biotechnology, sc-966; 1:500), a rabbit polyclonal anti-pSer33 (Bethyl, A300-246A; 1:2000), a mouse monoclonal anti-γH2AX (Millipore, 05-636; 1:2000) and a mouse monoclonal anti-Mus81 (Santa Cruz Biotechnology, sc-47692; 1:200). Secondary antibodies were Alexa Fluor 568-conjugated donkey anti-rabbit IgGs (Thermo Fisher Scientific, A10042; 1:1000) and Alexa Fluor 488-conjugated donkey anti-mouse IgGs (Thermo Fisher Scientific, A21202; 1.1000). Image acquisition and analysis were as for DNA FISH.

Statistical analysis

For direct comparison of two groups, we employed a paired two-tailed Student's t-test using Microsoft Excel or a nonparametric two-tailed Mann-Whitney U test using GraphPad Prism. Replicate numbers and *P* values are indicated in figure legends.

References

1. B. Silva, R. Arora, S. Bione, C. M. Azzalin, TERRA transcription destabilizes telomere integrity to initiate break-induced replication in human ALT cells. *Nat Commun* **12**, 3760 (2021).
